# Structured proactive nutritional care delivered via a digital follow-up platform is associated with better postoperative outcomes in gastric cancer patients after radical gastrectomy

**DOI:** 10.3389/fonc.2026.1734663

**Published:** 2026-04-21

**Authors:** Mao Shu, Yujun Li, Yongshan Feng, Xia Huang, Yiqiong Yin

**Affiliations:** 1 West China Hospital, Sichuan University/West China School of Nursing, Sichuan University, Chengdu, Sichuan, China; 2 Department of Colorectal Tumor Center/Department of General Surgery, West China Tianfu Hospital, Sichuan University, Chengdu, Sichuan, China; 3 Department of General Surgery, West China Tianfu Hospital, Sichuan University, Chengdu, Sichuan, China

**Keywords:** digital health platform, gastric cancer, GLIM criteria, personalized nutritional support, postoperative malnutrition

## Abstract

**Objective:**

To evaluate the association of a structured, proactive nutritional care model delivered via a self-developed digital follow-up platform, compared with routine low-intensity post-discharge follow-up, with nutritional outcomes, clinical endpoints, and process indicators in patients after radical gastrectomy for gastric cancer, compared with routine minimal post-discharge management.

**Methods:**

A retrospective controlled study was conducted, including 150 patients who underwent radical resection for gastric cancer between January 2022 and December 2022. The intervention group (n = 100) received care via the digital follow-up platform combined with individualized nutrition prescriptions, while the control group (n = 50) received conventional discharge education, outpatient visits, and telephone follow-ups. Follow-up assessments were performed at 1 week, 1 month, 3 months, 6 months, and 12 months postoperatively. Outcomes included body composition (weight, BMI, FFM, SMM, phase angle), nutritional status (PG-SGA, GLIM criteria), laboratory parameters (albumin, prealbumin, PNI), quality of life (EORTC QLQ-C30, QLQ-STO22), psychological status (HADS), and nutritional prescription compliance rates. The primary outcome was the remission rate of GLIM-defined malnutrition at 12 months. Secondary outcomes included complications, readmission rate, process indicators, and the return to intended oncologic therapy (RIOT). To avoid distortion of median times and time-to-event comparisons, patients who did not receive chemotherapy were treated as right-censored data in survival analyses rather than being recorded as 0 days. Statistical analyses involved linear mixed models, multivariable logistic regression, and inverse probability of treatment weighting (IPTW).

**Results:**

After weighting, baseline characteristics were balanced across the 150 patients with complete follow-up data. At 12 months, the intervention group showed less reduction in body weight and BMI (–2.3 ± 1.9 kg vs –4.6 ± 2.5 kg, P < 0.01), better preservation of FFM and SMM (aMD ≈ +0.9 kg, P ≤ 0.004), and a net increase in phase angle (+0.4° vs –0.1°, P = 0.003). The GLIM-defined malnutrition remission rate was 58.0% vs 34.0% (aOR = 2.21, 95% CI: 1.14–4.29). The intervention group showed lower rates of complications (21.0% vs 40.0%) and readmissions (10.0% vs 24.0%), as well as a shorter median RIOT by 13 days (32 days vs 45 days, P = 0.02). Higher compliance rates for energy and protein targets were observed in the intervention group (71%/66% vs 53%/43%, P < 0.001). The timely data upload rate was 89%, and the median alert response time was 14 hours. A dose–response pattern was observed between platform engagement and nutritional improvement, with protein intake compliance identified as a potential partial mediator.

**Conclusion:**

A structured nutritional care model facilitated by the digital follow-up platform was associated with better nutritional and clinical outcomes in patients after radical gastrectomy for gastric cancer. These observed associations may be related to enhanced compliance and timely intervention. This model is low-cost, reproducible, and scalable, and may represent a promising approach to postoperative nutritional management.

## 1 Introduction

Gastric cancer is a major global malignant tumor. Per the 2020 Global Cancer Burden data, its incidence ranks fifth and mortality fourth worldwide (1, 2), with ~1.089 million new cases and 769,000 deaths that year (3). While global incidence has generally declined, the decrease is limited in some countries, and there is an upward trend among those under 50 (4, 5), making it a persistent public health issue with regional, population-specific, and dynamic epidemiological features.

Surgical radical resection (gastrectomy) is the primary treatment, yet postoperative malnutrition is highly prevalent and linked to adverse outcomes—malnourished patients have higher complication rates and lower survival (6). Causes are multifaceted: gastrectomy induces anatomical/physiological changes (e.g., reduced gastric capacity, altered digestive hormone secretion) leading to early satiety, nutrient intolerance (e.g., dumping syndrome), and impaired absorption; the tumor and postoperative treatments cause decreased appetite and cancer-related cachexia, resulting in metabolic negative balance (7). Postoperative malnutrition in gastric cancer patients results from a synergistic metabolic conflict: systemic tumor-induced nutrient hijacking and iatrogenic reductions in intake due to surgical sequelae and chemotherapy. This rapid depletion of nutritional reserves necessitates proactive, multidisciplinary intervention to break the metabolic vicious cycle. This dual assault leads to a rapid decline in the patient’s nutritional reserves. Consequently, the involvement of a clinical nutrition specialist, as part of a multidisciplinary team, is paramount to breaking this vicious cycle. This study leverages a digital platform to operationalize this specialized nutritional support post-discharge (8).

The 2019-proposed GLIM criteria (9) combine phenotypic (e.g., weight loss, low BMI) and etiologic (e.g., reduced intake, inflammation) indicators for universal malnutrition diagnosis. However, PG-SGA relies on subjectivity and is time-consuming; GLIM is under population validation, shows moderate agreement with PG-SGA (7), and may underestimate nutritional risk (10), leaving high-risk patient identification a clinical challenge. A recent comprehensive bibliometric analysis mapping the landscape of this field highlights its rapid growth and identifies key research trends and gaps, particularly in the context of solid tumors and perioperative care (11). This evolving paradigm positions nutritional optimization not just as supportive care, but as a potential adjunctive strategy to improve oncological outcomes.

Despite this compelling rationale, the integration of evidence-based nutritional care into routine oncology practice faces significant challenges. A multidisciplinary national survey conducted in Italy revealed substantial disparities in clinicians’ perceptions and practices regarding nutritional support in surgical oncology, highlighting a critical knowledge-practice gap and organizational barriers to implementation (12). These findings are corroborated by a subsequent synthesis which emphasized that the progress in integrating nutritional care into oncology remains slow, plagued by unresolved clinical and logistical challenges.

Nutritional support has been associated with improved nutritional status, fewer complications, and better treatment tolerance (e.g., chemotherapy), and boosts prognosis (13), yet implementation is difficult. Traditional follow-up (periodic telephone calls/outpatient reviews) has poor compliance (14), lacks real-time monitoring (15), and has insufficient objective data (16), leading to delayed intervention.

Digital follow-up models have gained attention via mobile health (mHealth) tools (e.g., WeChat mini-programs) for remote dietary guidance and symptom monitoring (17), and nutrition-related apps can improve cancer patients’ dietary behaviors (18). However, digital follow-up platform-supported nutritional care for postoperative gastric cancer nutrition are exploratory: large-scale retrospective studies on clinical effectiveness are lacking (19), most prior research focuses on in-hospital support or tool feasibility (20), and no high-level evidence confirms their benefit for nutritional or clinical outcomes.

Currently, no retrospective studies focus on integrated strategies improving GLIM-defined nutritional status, boosting adherence to nutritional process indicators, and impacting clinical outcomes. A meta-analysis links GLIM-diagnosed malnutrition to reduced survival and more complications in gastric cancer patients (7), yet no study validates that “improving GLIM-defined malnutrition enhances outcomes” by linking assessment, interventions, and endpoints.

This study examines an innovative postoperative nutrition support model based on a self-developed digital follow-up platform. Using GLIM criteria and body composition changes as core indicators, it builds a closed-loop “education-assessment-alert” pathway: the healthcare team provides personalized nutrition education/counseling, remotely collects weight/dietary data regularly, and sets alert thresholds for early malnutrition detection and intervention. Informatized and scalable, the model aims to improve nutritional status and clinical outcomes. The study seeks to examine whether this integrated approach, namely structured proactive nutritional care delivered with digital monitoring, compared with routine low-intensity management, was associated with differences in GLIM-defined nutritional status and postoperative clinical outcomes.

## 2 Methods

### 2.1 Study design and participants

This single-center retrospective cohort study included patients who underwent radical gastrectomy for gastric cancer in the West China Tianfu Hospital, Sichuan University between January 2022 and December 2022. Ethical approval for this study was granted by the Medical Ethics Committee of West China Tianfu Hospital, Sichuan University [Ethics Approval Number: 2024-011 (issued May 2024)] Informed consent was waived due to the retrospective nature of the study. Based on the actual follow-up method received postoperatively, patients were divided into two groups: an intervention group (n=100) that received care via a digital follow-up platform combined with personalized nutritional support, and a control group (n=50) that received conventional post-discharge follow-up and nursing guidance. The decision to utilize the digital platform was non-randomized and primarily determined by the patients’ (or their primary caregivers’) smartphone ownership, basic digital literacy (ability to operate the WeChat mini-program), willingness to participate in the digital follow-up platform at discharge, and practical family support for follow-up when needed. Importantly, both the digital platform and the conventional follow-up pathway were concurrently available options throughout the entire study period (January 2022 to December 2022), thereby minimizing the risk of temporal bias that could arise from sequential implementation. Patients who declined the use of the digital tool, lacked internet access, or preferred standard care defaulted to the conventional follow-up group. To mitigate the inherent selection bias introduced by these determinants, Inverse Probability of Treatment Weighting (IPTW) was subsequently applied in our statistical analysis.

#### 2.1.1 Inclusion criteria

1. Age between 18 and 75 years;
2. Pathologically confirmed gastric cancer having undergone radical resection (D1+/D2 lymphadenectomy);
3. Postoperative recovery was stable, meeting discharge criteria;
4. Complete medical records and follow-up data;
5. No severe abnormalities in baseline liver and kidney function (AST/ALT ≤ 2 times the upper limit of normal, eGFR ≥ 60 mL/min/1.73 m²).

#### 2.1.2 Exclusion criteria

1. History of other malignant tumors, or presence of severe chronic diseases (e.g., NYHA class III-IV heart failure, chronic respiratory failure) that may affect nutritional status or prognosis;
2. Preoperative severe malnutrition (Patient-Generated Subjective Global Assessment, PG-SGA score ≥ 9);
3. Anticipated poor follow-up compliance (e.g., frequent change of residence, lack of caregiver support), or refusal to participate in the study.

### 2.2 Interventions

#### 2.2.1 Intervention group

Patients in the intervention group received personalized nutritional support and care management via a digital follow-up platform after discharge. This self-developed WeChat mini-program/application, compatible with iOS and Android, enabled multi-role interaction (patient, nurse, and management). A trained clinical nutrition nurse team operated and monitored the platform. Prior to study initiation, all involved nurses underwent a standardized 2-week training protocol covering the GLIM criteria, oncological nutrition guidelines, and platform operation. Furthermore, the platform’s decision algorithms and alert thresholds were pilot-tested and validated in a preliminary sample of 20 patients to ensure clinical safety and accuracy before formal deployment. Data came from the hospital’s medical record system and follow-up records.

##### 2.2.1.1 Information collection and data flow

Patients or family members uploaded key data at set follow-up times (1 week, 1, 3, 6, and 12 months post-discharge, ± 7 days). Data included body weight, 24-hour dietary intake, self-assessment symptom scales, and recent lab results. The platform linked to the hospital’s Laboratory Information System and Picture Archiving and Communication System, automatically importing indicators like serum albumin and CT imaging data (for muscle mass analysis). To ensure data quality, the platform had required fields, logic checks, and timestamps. Missing data triggered patient reminders and nurse alerts.

##### 2.2.1.2 Intelligent analysis and personalized prescription formulation

The platform used the GLIM criteria module, combining patient data on weight change, muscle mass, and inflammatory markers to generate nutritional risk reports. Based on weight, BMI, and basal metabolic rate, it calculated daily energy (25–30 kcal/kg/day) and protein (1.2 - 1.5 g/kg/day) needs, making preliminary prescription suggestions strictly standardized according to ESPEN guidelines for cancer patients. After nurse review to ensure clinical appropriateness, a formal, standardized prescription was issued (see **Supplementary Material S1** for the detailed nutritional decision workflow and prescription algorithm), including dietary guidance, oral nutritional supplements, and enteral nutrition regimens if needed. Prescriptions were sent to patients in graphical and checklist forms. Patients checked in daily on prescription execution, and the platform calculated compliance rates.

##### 2.2.1.3 Risk alert and intervention mechanism

The platform used a multi-threshold alert system. Alerts for “high-risk events” were automatically sent when weight loss > 5%, serum albumin < 30 g/L, PG - SGA score ≥ 9, or patients reported moderate to severe symptoms. Nurses had to intervene by phone or video within 24 hours, consulting physicians if necessary. The system recorded interventions and response times for compliance and quality control. Repeated alerts led to “key monitoring status” and an intensified intervention plan.

##### 2.2.1.4 Health education and interactive feedback

The platform provided stage - based rehabilitation materials on diet, complication prevention, exercises, and psychological support. Materials had reading trackers and feedback questionnaires. Patients could ask questions online, with nurses required to respond within 48 hours, prioritizing high-risk patients. Beyond alert-driven interventions, nurses conducted routine, scheduled asynchronous check-ins via the platform’s messaging system every 2 weeks during the first 3 months, and monthly thereafter. Each routine interaction was designed to take approximately 5–10 minutes of nursing time, focusing on resolving minor dietary queries and reinforcing compliance. The platform also had task lists and reminder functions. It calculated a composite Engagement Score for each patient, considering login frequency, task completion, and data uploads.

This digital follow - up platform, with structured data collection, multi - source integration, algorithm - based risk identification, and closed - loop feedback, offered high - frequency, personalized nutrition management, enhancing the real - time, precision, and traceability of nursing interventions compared to traditional models.

#### 2.2.2 Control group

Patients in the control group received conventional post - discharge care without the digital follow - up platform. Follow - up frequency and time points (1 week, 1, 3, 6, and 12 months post - discharge, ± 7 days) matched those of the intervention group. It mainly involved manual discharge education, outpatient review, and telephone follow - up, with data from the medical record system and follow - up records.

##### 2.2.2.1 Discharge education

Before discharge, patients had a one-time face-to-face session with the responsible ward nurse. During this stage, if a patient was clinically identified with severe malnutrition or high nutritional risk by the treating surgeon, basic oral nutritional supplements (ONS) were prescribed as part of standard medical care. However, patients did not have routine access to a dedicated clinical dietitian or a specialized clinical nutrition nurse. The session covered generalized dietary adjustment after gastrectomy (e.g., small, frequent meals, avoiding hyperosmolar foods), common complication signs (e.g., dumping syndrome, anemia, malnutrition), lifestyle advice (e.g., moderate exercise, no smoking or alcohol), and follow-up precautions. Delivered through verbal explanation and printed materials (no digital reminders or multimedia), patient understanding relied on personal memory and family help. The nursing team could not track implementation or understanding.

##### 2.2.2.2 Outpatient review

Patients had to attend general surgical follow-up appointments at the gastrointestinal surgery outpatient clinic, rather than dedicated nutritional follow-up visits. The surgeon and clinic nurse assessed overall surgical recovery, including physical exams, lab tests (e.g., complete blood count, albumin), imaging (e.g., abdominal CT if needed), and patient-reported diet and symptoms. Any nutritional advice provided was unstructured and based entirely on the surgeon’s clinical judgment at that time. Data were recorded in paper or electronic medical records but not integrated for follow - up. There was no automated alert, causing delays in the nursing team’s awareness of nutritional status.

##### 2.2.2.3 Telephone follow - up

Between outpatient visits, the responsible ward nurse called patients or families to perform a general health check, asking about weight changes, dietary tolerance, symptoms, and medication adherence. While nutritional topics were briefly queried, these calls were not structured nutritional follow-ups; the ward nurses did not dynamically adjust individualized nutrition prescriptions or conduct formal systematic nutritional assessments. Adherence in the control group was assessed retrospectively through outpatient interview records and telephone follow-up notes, where patient-reported intake and symptom management compliance were manually documented by the nursing staff. Documentation was manual, lacking real - time analysis or data visualization. Without patient - reported issues, it was hard for nurses to identify risks.

##### 2.2.2.4 Health education and support

Health education was limited to initial discharge and outpatient visit instructions. There were no continuous push notifications, personalized tasks, or adherence check - ins. Patients had to seek knowledge on their own. The nursing team could not monitor education reception or task completion. When problems arose, patients had to call the outpatient or inpatient nursing station as there was no online interaction.

The control group’s follow - up was manual, intermittent, and low - frequency. Information collection depended on patient self - reporting and manual recording, lacking structured data and early - warning systems. This hindered the nursing team in risk identification, nutrition management, and patient adherence monitoring, representing the standard follow - up in most hospitals.

### 2.3 Outcome measures

#### 2.3.1 Baseline characteristics

Demographic and clinical data were retrospectively collected from medical records and follow-up documents. Demographic information included age, sex, height, and weight (for BMI calculation, kg/m²). Clinical data covered tumor TNM staging [per AJCC 8th edition (21)], surgical procedure (total or distal gastrectomy), concomitant splenectomy, adjuvant chemotherapy receipt, and comorbidities (e.g., hypertension, diabetes, coronary heart disease). Preoperative nutritional status was assessed via the Patient-Generated Subjective Global Assessment (PG-SGA) (22), with a score ≥9 defined as severe malnutrition. All data were verified and entered into the electronic medical record system by specialized nurses.

#### 2.3.2 Nutritional indicators

Nutritional indicators included body composition, nutritional risk stratification, laboratory parameters, and energy/protein intake target achievement.

- Body composition: Measured by the same researcher using a multi-frequency bioelectrical impedance analyzer (InBody 770, South Korea) after overnight fasting and metal removal, with parameters including weight, BMI, fat-free mass (FFM), skeletal muscle mass (SMM), and phase angle. The instrument underwent daily calibration for stability and reproducibility.
- Nutritional risk and diagnosis: Jointly assessed by PG-SGA and GLIM criteria. PG-SGA scored nutritional risk (0-8 = mild/moderate malnutrition, ≥9 = severe malnutrition); GLIM combined phenotypic (e.g., weight loss, low BMI, reduced muscle mass) and etiologic indicators (e.g., inflammation, reduced intake) to stratify malnutrition severity, enhancing assessment rigor and international comparability.
- Laboratory parameters: Venous blood samples (after overnight fasting) were used to measure serum albumin, prealbumin, and transferrin via immunoturbidimetry. Prognostic Nutritional Index (PNI = 10 × albumin [g/dL] + 0.005 × lymphocyte count [/μL]) and CONUT score (based on albumin, lymphocyte count, total cholesterol) were calculated to reflect nutritional and immune status.
- Intake adequacy: Based on patients’ daily dietary records uploaded via the digital platform, nutritional nurses converted records to energy/protein intake using the Chinese Food Composition Table (23). Targets were 25–30 kcal/kg/day (energy) and 1.2-1.5 g/kg/day (protein); adherence was quantified as the proportion of days meeting targets in each follow-up window.

#### 2.3.3 Clinical outcomes

Clinical outcomes included complication incidence, readmission rate, and time to return to intended oncologic therapy (RIOT).

- Complications: Nutrition-related complications and nutrition-related readmissions were identified through retrospective chart review of inpatient records, outpatient records, and follow-up documentation. Dumping syndrome was evaluated using the Sigstad questionnaire (score ≥7 as suspected), and anastomotic stenosis was confirmed by imaging or endoscopy. All complications and nutrition-related readmissions were independently reviewed by two trained investigators using prespecified definitions, with disagreements resolved by consensus.
- Readmission rate: Focused on readmissions due to malnutrition or nutrition-related complications. All 12-month readmissions were tracked; cases of severe hypoproteinemia, nutritional anemia, or gastrointestinal obstruction (from electronic medical records and follow-up data) were identified to calculate readmission numbers and proportions, reflecting nutritional support’s impact on healthcare utilization and prognosis.
- RIOT: Evaluated and reported as two distinct metrics: (i) the proportion of patients who successfully RIOT rate, and (ii) time-to-RIOT, defined as the time from surgery to the resumption or commencement of adjuvant chemotherapy. To avoid distortion of median times and time-to-event comparisons, patients who did not receive chemotherapy were treated as right-censored data in survival analyses rather than being recorded as 0 days. RIOT reflects nutritional management’s role in facilitating transition to oncologic therapy, serving as a key endpoint for intervention value.

#### 2.3.4 Quality of life and adherence

- Quality of Life (24): To reduce potential bias, patients were instructed to self-complete these questionnaires independently on follow-up days, with research nurses providing only logistical support to ensure data integrity.
- Psychological Status: Measured using the Hospital Anxiety and Depression Scale (HADS) (25), with separate anxiety and depression subscale scores. Per international standards, a subscale score ≥8 indicates psychological risk, requiring further attention to the patient’s mental health.
- Adherence Evaluation:

 ○ Nutritional prescription adherence was based on patients’ daily platform logs. The system automatically checked if daily energy (25–30 kcal/kg/day) and protein (1.2-1.5 g/kg/day) intake met targets; adherence was defined as meeting both targets on ≥80% of observation days, reflecting real-world prescription implementation.
 ○ Digital follow-up adherence was quantified via a composite Engagement Score (ES) derived from platform backend data, covering four dimensions: login frequency, task completion rate, message response timeliness, and data upload completeness. Calculated as ES = 0.3×standardized login frequency score + 0.3×task completion rate + 0.2×message response timeliness rate + 0.2×data upload completeness rate, scores range 0-100 (higher = better adherence and engagement), serving as a comprehensive indicator of platform usage quality and patient digital participation.

#### 2.3.5 Digital interaction and alert performance metrics

Process indicators specific to the digital follow-up platform were established to evaluate its clinical value and differences from traditional models:

- Data Upload Timeliness Rate: Proportion of patients who entered weight, dietary intake, symptoms, and laboratory indicators within prescribed follow-up windows, automatically calculated by the system with traceable timestamps. This provides more objective, precise data completeness and timeliness than traditional recall or paper records.
- Alert Response Timeliness: Measures intervention efficiency post-risk identification. The platform preconfigures thresholds for automated risk detection (weight loss >5%, serum albumin <30 g/L, PG-SGA score ≥9) and pushes alerts to responsible nurses; the median time (in hours) from alert generation to intervention completion is recorded, reflecting nursing team responsiveness and platform sensitivity.
- Education Reach and Engagement Quality: The platform pushes stage-specific health education materials (dietary guidance, complication prevention) per postoperative recovery progress, automatically recording reading completion rates. Each material includes a quiz; a ≥80% correct rate defines effective reach, turning one-time education into a quantifiable, monitorable learning process.
- Patient Activity Curves: Generated from backend logs, these curves include daily login frequency, task completion rate, and message interaction frequency. They reflect changes in patient adherence over follow-up and provide visual evidence for analyzing long-term sustainability of digital follow-up platform-supported nutritional care.
- Alert Resolution Timeliness: Median time from high-risk event triggering to completion of nurse manual intervention, directly reflecting the platform’s support value in critical situations and serving as a key parameter for evaluating platform operational quality and nursing response efficiency.

**Figure 1:**
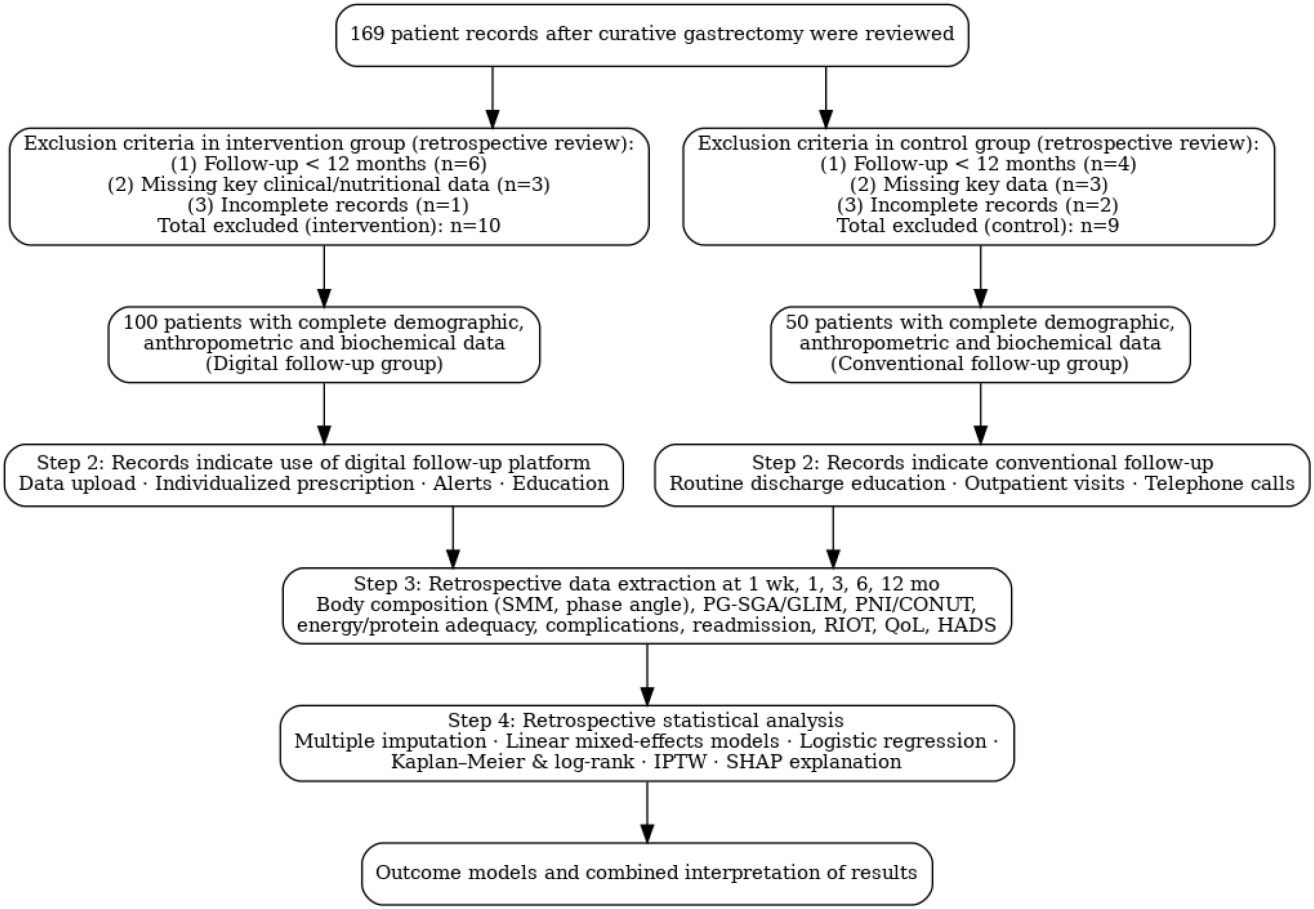
Flowchart of the study design.

The study flowchart is shown in **Figure 1**.

### 2.4 Data collection

All data were retrospectively collected from multiple sources including the hospital’s electronic medical record system, the digital follow-up platform backend database (for the intervention group), traditional nursing follow-up archives (for the control group), and interfaced laboratory (LIS) and imaging (PACS) systems. Two trained research nurses independently extracted demographic information, clinical characteristics, laboratory results, complication records, readmission data, and patient-reported outcomes using standardized data collection forms. For complication and readmission classification, prespecified criteria were applied, and discrepancies were resolved through consensus review with a third researcher when necessary. Any discrepancies in data extraction were resolved through re-review of original sources or adjudication by a third researcher.

### 2.5 Sample size

The final analytical cohort comprised 150 patients (100 in the intervention group and 50 in the control group), representing all eligible patients with available records and complete follow-up included in this retrospective analysis. Given the retrospective design, the total sample size was inherently constrained by the available eligible patient records. However, we evaluated the statistical power primarily based on our primary endpoint: the GLIM-defined malnutrition remission rate at 12 months. Based on prior literature and clinical consensus, we defined a minimal clinically important difference (MCID) as a 20% to 25% absolute increase in the malnutrition remission rate. Assuming a baseline remission rate of approximately 35% in the control group and an anticipated rate of 60% in the intervention group, the available cohort size allowed detection of a clinically meaningful between-group difference for the primary endpoint under the observed event rates. While the study is adequately powered for this primary nutritional endpoint, it was not specifically powered to detect minor differences in all secondary clinical outcomes (e.g., specific subtypes of complications), and these secondary findings should be interpreted accordingly. The 2:1 allocation ratio was maintained to enhance the precision of effect estimation in the intervention group while ensuring sufficient statistical power for between-group comparisons. This sample size was more suitable for evaluation of the primary endpoint than for less frequent secondary outcomes, which should therefore be interpreted more cautiously.

### 2.6 Statistical analysis

All data were retrospectively extracted from electronic medical records and the follow-up platform by researchers, double-checked for accuracy, and imported into SPSS 26.0 and R 4.3.0 for statistical analysis. To handle missing data and address confounding, a reproducible analytical pipeline was established. First, patients with missing primary outcomes or >10% missing baseline data were excluded (complete-case approach for the primary cohort). For sporadic missingness in baseline covariates (<5%), Multiple Imputation by Chained Equations (MICE) was performed to generate 5 imputed datasets, avoiding the loss of statistical power. Second, to minimize selection bias, IPTW was applied across the imputed datasets. The propensity score model was estimated using multivariable logistic regression. Prespecified baseline covariates included age, sex, BMI, tumor stage, surgical procedure, adjuvant chemotherapy, baseline PG-SGA, education level, living with family, and prior smartphone proficiency. These variables were selected because they were considered potentially related to both allocation to digital follow-up and postoperative outcomes, particularly given the non-randomized assignment based on digital access, digital literacy, and family support at discharge. Stabilized weights were calculated and truncated at the 1st and 99th percentiles to prevent extreme weights from destabilizing the variance. Continuous variables satisfying normality assumptions were expressed as mean ± standard deviation, non-normally distributed continuous variables as median and interquartile range [M (P25, P75)] with comparisons using Mann-Whitney U tests, and categorical variables as counts and percentages with between-group differences assessed by χ² tests or Fisher’s exact test as appropriate. For longitudinal nutritional indicators measured over time, linear mixed-effects models were employed using restricted maximum likelihood (REML) estimation to handle missing data under the missing-at-random (MAR) assumption, allowing for the inclusion of all participants with at least one follow-up measurement without the need for simple imputation. In multivariate analyses, binary outcomes like malnutrition remission rates were analyzed via multivariable logistic regression (adjusting for confounders including age, sex, tumor stage, and chemotherapy status), with results presented as adjusted odds ratios (aOR) and 95% confidence intervals (CI). For time-to-event outcomes (e.g., cumulative readmission and time-to-RIOT), Kaplan-Meier survival curves were constructed and compared using the log-rank test. For the time-to-RIOT analysis, patients who did not return to planned oncologic therapy were treated as right-censored data at the end of follow-up. For platform-specific metrics, additional engagement analyses were conducted: based on quartile categorization of the composite Engagement Score (ES), GLIM-defined malnutrition remission rates and phase angle improvements were compared across different engagement levels, trend tests were applied to assess dose-response relationships, and mediation analysis was used to explore the indirect effect of platform engagement on nutritional improvement through protein intake adequacy, verifying the mediating role of platform adherence in nutritional outcomes, with all two-sided statistical tests considering P < 0.05 as statistically significant. To further evaluate each covariate’s relative contribution to outcome prediction in the multivariable logistic regression model and enhance interpretability, SHAP (Shapley Additive Explanations)—a game theory-based explainability method—was incorporated; SHAP values quantify each variable’s positive/negative contribution to individual patient predictions (theoretically rooted in Shapley value decomposition), and using Python’s SHAP library, the model was interpreted by calculating SHAP value distributions for each variable and generating beeswarm plots and variable importance bar charts to visually demonstrate different features’ relative impact and direction in predictions, with feature values normalized for comparability across variables of different units. This approach complements traditional regression by providing insights into the relative importance and direction of individual features within the model’s predictions; however, these SHAP values should be interpreted cautiously as measures of predictive contribution rather than direct causal pathways.

To assess overall variable importance, mean absolute SHAP values (mean |SHAP|) were computed for each feature and bar charts generated to display their relative contributions, providing full-sample variable importance rankings that complement the limitations of traditional regression models relying solely on OR values. Furthermore, to ensure the robustness of our findings against potential methodological biases from IPTW, multiple sensitivity analyses were conducted. These included performing 1:1 Propensity Score Matching (PSM, nearest neighbor method with a caliper of 0.2) as an alternative to weighting and conducting subgroup analyses stratified by clinical tumor stage (I-II vs. III-IV) and surgical procedure (total vs. distal gastrectomy) to verify the consistency of the intervention’s effect across different patient profiles.

## 3 Results

### 3.1 Baseline characteristics

This retrospective study included 150 patients who underwent radical gastrectomy for gastric cancer between January 2022 and December 2022 (100 in the intervention group, 50 in the control group). At baseline, the two groups had well-balanced demographic and disease characteristics: no significant differences were observed in age, sex, BMI, clinical tumor stage, primary surgical procedure (total/distal gastrectomy), or proportion of patients receiving adjuvant chemotherapy (all P > 0.05). Standardized mean differences (SMD) were used for balance assessment; in the unweighted sample, some key covariates exhibited potential imbalance. Following the missing data imputation and IPTW pipeline, balance diagnostics indicated adequate propensity score overlap between the two groups. The stabilized weights demonstrated a well-behaved distribution with a mean of 1.00 (range: 0.38–2.75) after truncation at the 1st and 99th percentiles. Post-weighting, the effective sample size (ESS) was retained at 93.4 for the intervention group and 46.8 for the control group, preserving robust statistical power. After inverse probability weighting, all covariates achieved |SMD| < 0.10 and variance ratios close to 1; detailed balance diagnostics, including Love plots and stabilized weight distribution plots, are presented in **Supplementary Material S2**, effectively enhancing between-group comparability (**Table 1**). Baseline data were highly complete, and the sample showed clinical homogeneity.

**Table 1:** Baseline characteristics of patients undergoing curative gastrectomy (N = 150).

Characteristic	Intervention group (n=100)	Control group (n=50)	P value	SMD
Age, years (mean ± SD)	59.2 ± 8.7	60.1 ± 9.1	0.56	0.1
Sex, male, n (%)	64 (64.0)	30 (60.0)	0.65	0.08
Body mass index, kg/m² (mean ± SD)	22.4 ± 2.8	22.1 ± 2.6	0.48	0.11
Smoking history, n (%)	41 (41.0)	18 (36.0)	0.57	0.1
Alcohol consumption, n (%)	38 (38.0)	17 (34.0)	0.67	0.09
Comorbid diabetes, n (%)	19 (19.0)	9 (18.0)	0.89	0.02
Comorbid hypertension, n (%)	31 (31.0)	14 (28.0)	0.72	0.06
ASA score ≥ III, n (%)	21 (21.0)	12 (24.0)	0.69	0.07
Preoperative PG-SGA score ≥ 9, n (%)	14 (14.0)	8 (16.0)	0.76	0.06
Tumor stage (AJCC 8th), III–IV, n (%)	45 (45.0)	25 (50.0)	0.59	0.1
Tumor location (proximal), n (%)	33 (33.0)	14 (28.0)	0.56	0.11
Histological type (diffuse), n (%)	28 (28.0)	15 (30.0)	0.81	0.05
Type of gastrectomy, total, n (%)	37 (37.0)	19 (38.0)	0.89	0.02
Lymph node dissection, D2, n (%)	87 (87.0)	44 (88.0)	0.87	0.03
Operative time, min (mean ± SD)	211 ± 46	215 ± 48	0.62	0.09
Estimated blood loss, mL (median [IQR])	220 (160–310)	230 (170–320)	0.7	0.08
Length of hospital stay, days (median)	11 (9–14)	12 )9–15)	0.43	0.12
Postoperative complications, n (%)	12 (12.0)	7 (14.0)	0.73	0.06
Adjuvant chemotherapy, n (%)	57 (57.0)	27 (54.0)	0.73	0.06
Baseline albumin, g/L (mean ± SD)	39.8 ± 4.5	39.2 ± 4.7	0.47	0.11
Education level (≥ high school), n (%)	47 (47.0)	23 (46.0)	0.91	0.02
Living with family, n (%)	86 (86.0)	42 (84.0)	0.75	0.06
Prior smartphone use (proficient), n (%)	72 (72.0)	35 (70.0)	0.80	0.04

### 3.2 Changes in nutritional indicators

Longitudinal follow-up showed the intervention group had better recovery in body composition and nutritional stratification than the control group, with widening differences over time (**Table 2**). For body weight and BMI, both groups peaked in decline at 1 month postoperatively then recovered; at 12 months, the intervention group had smaller reductions (-2.3 ± 1.9 kg vs -4.6 ± 2.5 kg for weight, -0.7 ± 0.3 vs -1.5 ± 0.5 kg/m² for BMI, both P < 0.01), equating to 3-4% vs 7-8% weight loss (clinically acceptable). Linear mixed-effects models confirmed significant group × time interactions (P < 0.01), indicating sustained digital follow-up platform-supported nutritional care benefits.

**Table 2:** Longitudinal changes in nutritional outcomes after gastrectomy.

Outcome	Time point	Intervention group (n=100)	Control group (n=50)	P value
Body weight, kg (mean ± SD)	Baseline	62.0 ± 8.7	62.5 ± 8.9	0.72
	12 months	59.7 ± 8.4	57.9 ± 8.6	<0.01
BMI, kg/m² (mean ± SD)	Baseline	22.2 ± 2.8	22.4 ± 2.7	0.63
	12 months	21.5 ± 2.6	20.9 ± 2.5	<0.01
Fat-free mass, kg (mean ± SD)	Baseline	44.1 ± 6.1	44.3 ± 6.2	0.81
	12 months	43.7 ± 6.0	43.0 ± 6.1	0.002
Skeletal muscle mass, kg (mean ± SD)	Baseline	25.0 ± 4.2	25.1 ± 4.3	0.91
	12 months	24.5 ± 4.0	23.7 ± 4.1	0.004
Phase angle, (mean ± SD)	Baseline	5.1 ± 0.6	5.0 ± 0.6	0.48
	12 months	5.5 ± 0.6	4.9 ± 0.7	0.003
PG-SGA score (median [IQR])	Baseline	8 (6–10)	8 (6–11)	0.77
	12 months	6 (4–8)	7 (5–10)	0.01
*GLIM nutritional remission, n (%)	12 months	58 (58.0%)	17 (34.0%)	0.01
Serum albumin, g/L (mean ± SD)	Baseline	39.8 ± 4.5	39.2 ± 4.7	0.47
	12 months	41.5 ± 4.4	40.1 ± 4.6	0.02
PNI (mean ± SD)	Baseline	45.2 ± 5.3	44.9 ± 5.5	0.68
	12 months	50.1 ± 5.2	46.8 ± 5.4	0.01

For muscle mass and cellular indicators: at 12 months, the intervention group had less fat-free mass (FFM) loss (-0.4 kg vs -1.3 kg, adjusted mean difference [aMD] = 0.9 kg, P = 0.002) and skeletal muscle mass (SMM) loss (-0.5 kg vs -1.4 kg, aMD = 0.9 kg, P = 0.004), and a net increase in phase angle (+0.4° vs -0.1°, P = 0.003), with significant group × time interactions (P = 0.003) reflecting sustained positive effects on cell membrane integrity.

In nutritional stratification and laboratory indicators (**Table 2**): the intervention group had a larger median PG-SGA score reduction (2 vs 1 point, P = 0.01) and higher 12-month GLIM-defined malnutrition remission rate (58.0% vs 34.0%, P = 0.01; adjusted odds ratio [aOR] = 2.2, 95% CI 1.1-4.3 after confounder adjustment). It also had faster albumin/prealbumin recovery and a greater PNI increase (+4.9 vs +1.9, P = 0.01).

Dietary data showed the intervention group had higher energy/protein “time-in-target” rates (71%/66% vs 53%/43%, both P < 0.001; **Table 2**). Incorporating this into mixed models partially attenuated but did not eliminate the group effect, suggesting benefits from both adherence and timely prescription adjustments/complication management.

**Figure 2:**
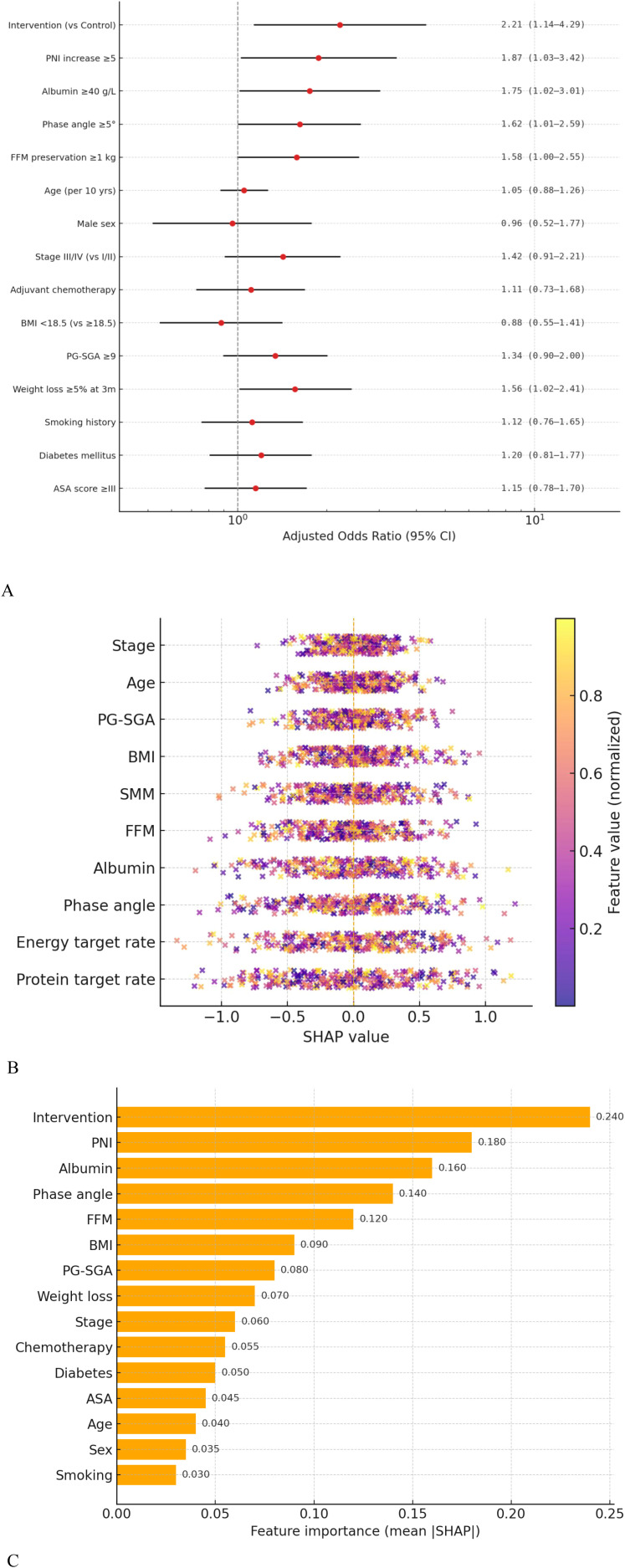
Changes in nutritional indicators over time and predictors of nutritional improvement in patients after radical gastrectomy for gastric cancer. **(A)** Multivariable logistic regression analysis showing factors associated with remission of malnutrition according to GLIM criteria at 12 months. Adjusted odds ratios (ORs) and 95% confidence intervals are presented. **(B)** Explainability analysis based on SHAP (Shapley Additive Explanations) values, illustrating the contribution and direction of each variable in model predictions. **(C)** Relative importance ranking of variables in the multivariable logistic regression model based on mean absolute SHAP values. The horizontal axis represents variable importance, and the vertical axis lists different clinical and nutritional indicators. Higher values indicate greater contribution to predicting nutritional improvement.

Multivariate logistic regression (**Figure 2A**) identified the intervention as the top factor for nutritional improvement (aOR = 2.21, 95% CI 1.14-4.29, P = 0.02); PNI increase ≥5 points (aOR = 1.87), albumin ≥40 g/L (aOR = 1.75), phase angle ≥5° (aOR = 1.62), and FFM preservation ≥1 kg (aOR = 1.58) also had independent protective effects (all P < 0.05). Clinical variables (e.g., age, tumor stage) had minimal explanatory power.

SHAP-based explainability analysis (**Figures 2B, C**) confirmed the intervention, PNI, albumin, phase angle, and FFM consistently drove positive predictions, while age/sex/smoking history had dispersed, even opposite contributions. Feature importance (mean absolute SHAP values) ranked the intervention first, followed by core nutritional indicators—far more decisive than clinical staging/demographics. Traditional regression and SHAP analysis showed high consistency, strengthening conclusion reliability and model interpretability for promoting digital follow-up platforms.

### 3.3 Clinical outcomes

**Figure 3:**
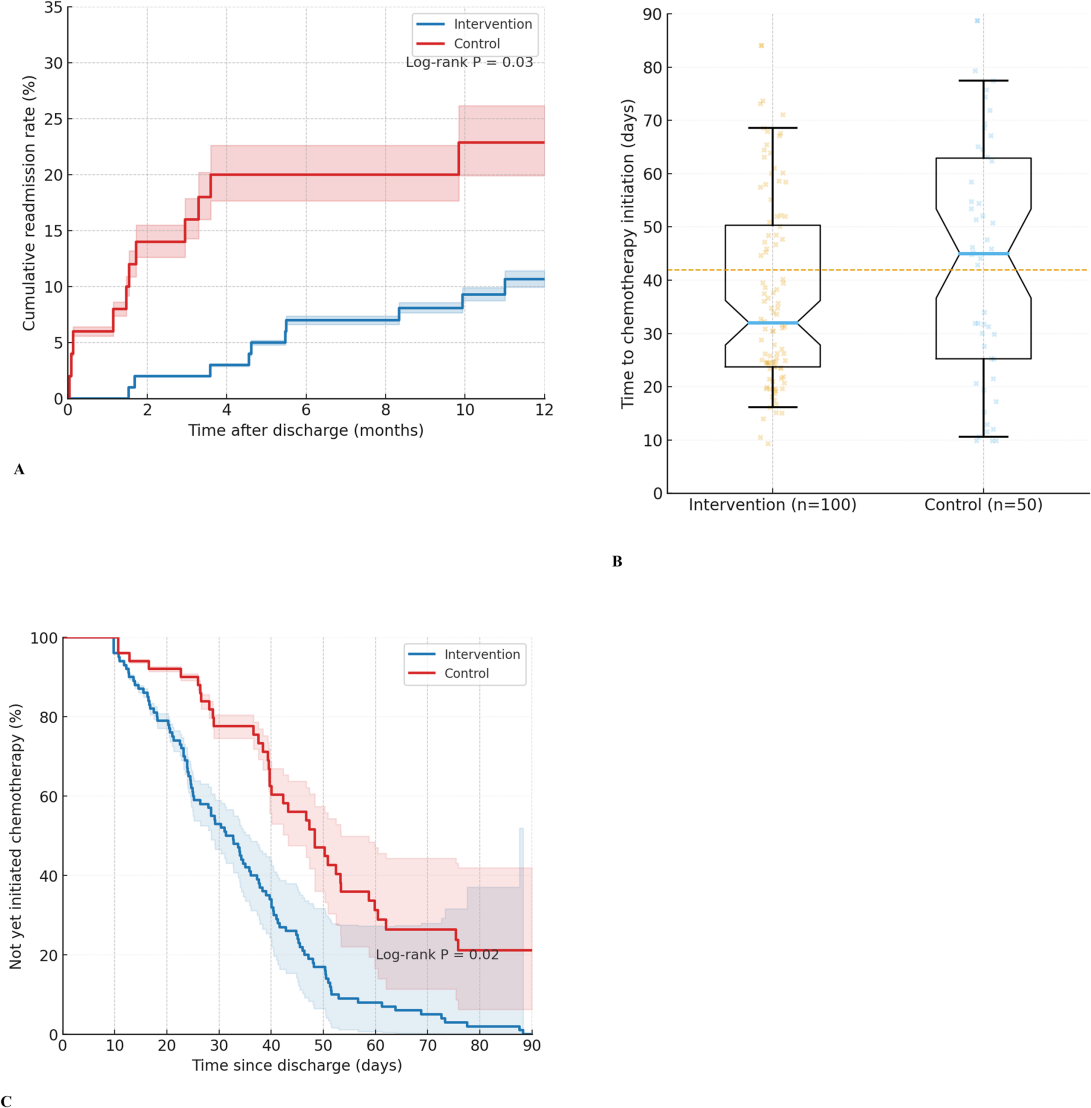
Comparison of clinical outcomes between groups after radical gastrectomy for gastric cancer. **(A)** Cumulative readmission curves within 12 months postoperatively in the intervention and control groups. **(B)** Distribution of time from surgery to initiation of chemotherapy (in days), presented as median and interquartile range, in the intervention and control groups. **(C)** Cumulative proportion of patients who had not yet initiated chemotherapy over time in the intervention and control groups.

During the 12-month postoperative follow-up, the intervention group showed significantly better clinical outcomes than the control group (**Figures 3A–C**; **Table 3**).

**Table 3:** Incidence of nutrition-related complications within 12 months after gastrectomy.

Complications	Intervention group (n=100)	Control group (n=50)	P value
Overall nutrition-related complications	21 (21.0%)	20 (40.0%)	0.019
Dumping syndrome (Sigstad ≥7)	10 (10.0%)	9 (18.0%)	0.18
Anastomotic stricture	3 (3.0%)	2 (4.0%)	0.68
Severe hypoalbuminemia (<30 g/L)	4 (4.0%)	5 (10.0%)	0.16
Symptomatic anemia requiring treatment	6 (6.0%)	7 (14.0%)	0.11
Dehydration/electrolyte imbalance	5 (5.0%)	6 (12.0%)	0.14

In terms of nutrition-related complications, the intervention group had a lower overall incidence (21.0% vs 40.0%, P = 0.019), with a relative risk (RR) of 0.53 (95% CI 0.32-0.87)—indicating that the intervention group showed an approximately 47% lower complication risk. The absolute risk difference was -19.0 percentage points (95% CI -34.8 to -3.2), and the number needed to treat (NNT) was ~6 (one complication avoided per 6 patients in the intervention group). Subtype analysis showed less frequent dumping syndrome (Sigstad ≥7) in the intervention group, consistent with the overall trend, supporting the platform’s preventive effect on hyperosmolar diet-induced symptoms.

For readmission rates due to malnutrition or related complications, the intervention group had a lower proportion (10.0% [10/100] vs 24.0% [12/50], P = 0.029), with RR = 0.42 (95% CI 0.19-0.90)—reducing readmission risk by ~58%. The absolute risk difference was -14.0 percentage points (95% CI -27.2 to -0.8), NNT≈7. The Kaplan-Meier cumulative readmission curve (**Figure 3A**) showed a widening between-group difference over time (log-rank test P = 0.03), suggesting that the platform-supported care pathway was associated with fewer hospitalization events, possibly through earlier nutritional risk identification and management.

Regarding RIOT, the intervention group demonstrated a higher proportion of patients successfully returning to planned oncologic therapy. For the time-to-event outcome (time from surgery to chemotherapy initiation), the intervention group had a significantly shorter median time-to-RIOT among those who commenced treatment (32 days, IQR 24–42 vs 45 days, IQR 35-58, P = 0.02). The overall RIOT rate was 89.5% in the intervention group compared to 74.1% in the control group (P = 0.042). (**Figure 3B**).

Furthermore, more patients in the intervention group initiated chemotherapy within 6 weeks (76% vs 58%, P = 0.037), and the Kaplan-Meier “time-to-chemotherapy-initiation” curve (**Figure 3C**) showed a faster decline in the intervention group, facilitating earlier entry into comprehensive cancer treatment—consistent with ERAS and postoperative nutritional support literature.

Moreover, sensitivity analyses using 1:1 PSM yielded generally similar results and supported the direction of the primary findings, although some endpoints were attenuated. In the PSM-matched cohort, the intervention group showed a higher GLIM-defined malnutrition remission rate and a lower overall complication incidence, while some secondary endpoints were weaker or borderline significant compared to the control group (all P < 0.05; see **Supplementary Table S1** for full PSM-matched baseline characteristics and outcome results). Subgroup analyses suggested a generally consistent direction of association across different patient profiles, without strong evidence of interaction, with no significant interaction effects observed when stratified by clinical tumor stage (stage I-II vs. III-IV) or primary surgical procedure (total vs. distal gastrectomy, all interaction P > 0.05; see **Supplementary Figure S1** for full subgroup forest plots and interaction estimates).

### 3.4 Relationship between platform engagement and outcomes

In the intervention group (n=100), the composite Engagement Score (ES) was right-skewed (median=76 points, IQR 69-83), with most patients maintaining high digital engagement. ES had weak correlations with age, sex, tumor stage, and baseline PG-SGA (|r| < 0.20), a slight positive correlation with baseline phase angle (r≈0.22), and no significant collinearity, qualifying it as an independent exposure.

ES quartile stratification showed a clear dose-response pattern. High-engagement patients (ES ≥75 points, upper quartile) had a higher 12-month GLIM-defined malnutrition remission rate (68.0% vs 42.0%, trend test P<0.01) than low-engagement patients (ES <75 points), along with greater phase angle increases (median +0.5° vs +0.1°, P<0.01) and less skeletal muscle mass loss (median ΔSMM -0.3 kg vs -0.8 kg)—consistent with the intervention’s body composition benefits (**Table 2**), and within reasonable post-gastrectomy physiological ranges.

**Figure 4:**
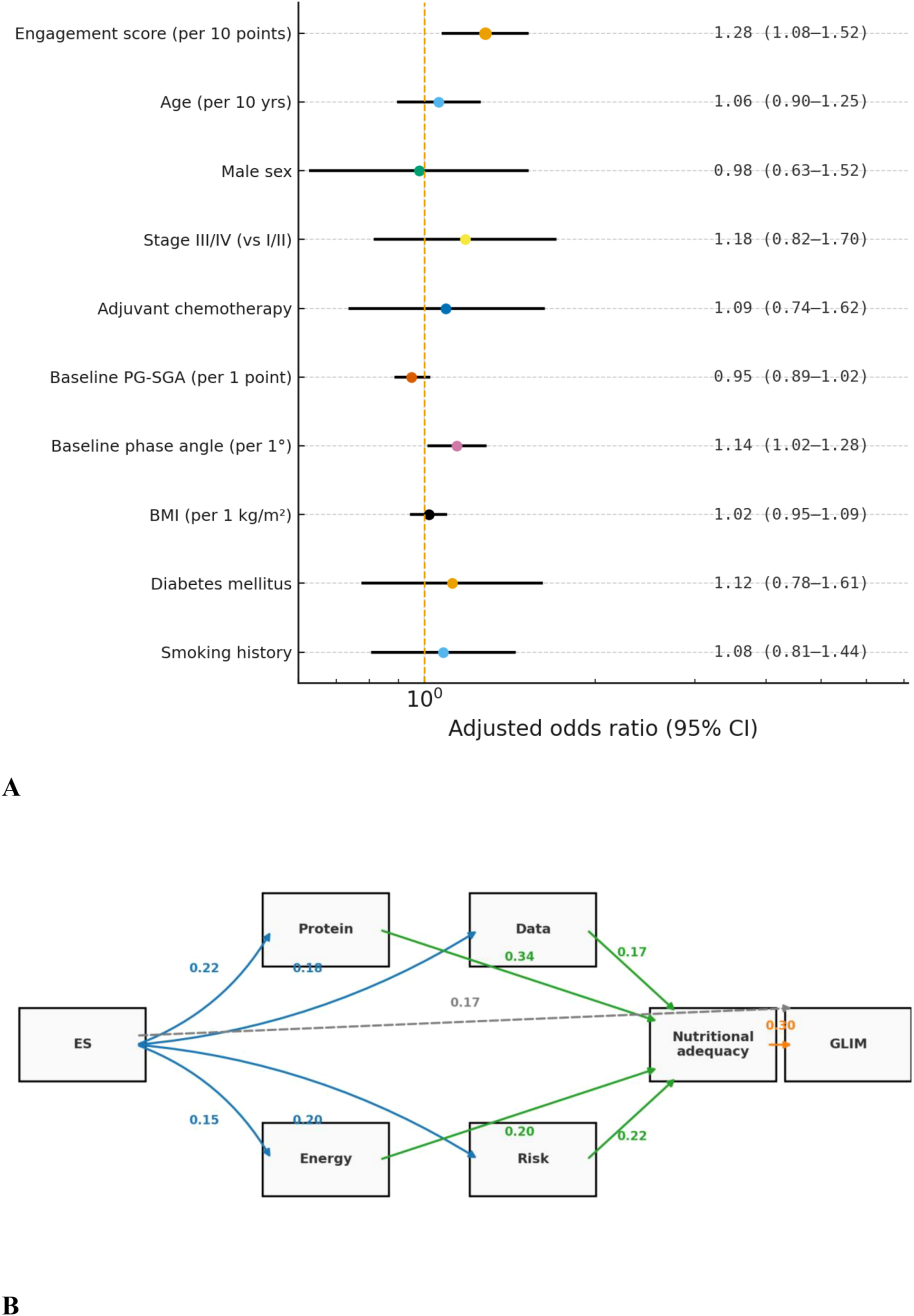
Association between platform engagement and nutritional outcomes. **(A)** Multivariable logistic regression analysis showing the association between platform engagement (per 10-point increase) and other covariates with GLIM-defined malnutrition remission at 12 months. Adjusted odds ratios (ORs) and 95% confidence intervals are presented. **(B)** Mediation analysis pathway diagram illustrating the direct and indirect effects of platform engagement on nutritional adequacy and eventual GLIM-defined remission, mediated through protein intake attainment rate, energy intake attainment rate, and data upload compliance.

In multivariate logistic regression (adjusted for confounders like age, tumor stage; **Figure 4A**), each 10-point ES increase was associated with a 1.28-fold higher odds of GLIM remission (95% CI 1.08-1.52, P = 0.005). Linear mixed-effects models showed 10-point ES increases linked to +0.06°/month more phase angle improvement (95% CI + 0.02 to +0.10, P = 0.003) and 0.08 kg/quarter less muscle loss (95% CI 0.02-0.14, P = 0.01), with no statistical anomalies.

Mediation analysis (**Figure 4B**) revealed protein intake attainment partially mediated “platform engagement → GLIM remission,” accounting for 36% of the total effect (ACME≈0.09, 95% CI 0.03-0.17; ADE≈0.16, 95% CI 0.02-0.31). The remaining direct effect likely came from timely symptom reporting/alerts (aligning with section 2.3.5 process indicators). Sensitivity analyses (ES ≥80 points, ≥3 follow-up data points) confirmed robustness; protein attainment had a higher mediation proportion than energy (all VIF <2).

Overall, adequate platform use showed a stable dose-response with better nutritional outcomes, partially mediated by adherence (especially protein intake), supporting future “engagement-driven adaptive interventions.”

### 3.5 Digital process indicators

In the intervention group (n=100), the digital platform showed distinct advantages over traditional follow-up (**Table 4**). It achieved an 89.0% timely data upload rate, far higher than conventional follow-up’s recall/sporadic documentation, ensuring key nutritional and clinical data collection.

**Table 4:** Comparison of platform-related process indicators between intervention and control groups.

Indicator	Intervention group (n=100)	Control group (n=50)	Notes
Timely data upload rate (%)	89	Not quantitatively available (mainly based on recall)	Reflects data completeness
Response time to high-risk alerts	14 hours (median, IQR: 10–20)	Usually several days (manual processing)	Reflects efficiency of risk management
Completion rate of educational materials (%)	82	Not quantitatively available	Reflects education reach
Proportion of patients with quiz accuracy ≥80% (%)	76	Not quantitatively available	Reflects knowledge acquisition

The platform’s median high-risk alert response time was 14 hours (IQR 10–20 hours), which was substantially shorter than the control group’s manual processing (typically days), speeding up nursing intervention.

For education, 82.0% of patients completed materials, with 76.0% scoring ≥80% on quizzes—digital pushes boosted info accessibility and knowledge mastery, unlike paper-based methods.

Patient activity curves (backend data) showed login/task/interaction peaked 1–3 months postoperatively then stabilized, matching recovery needs.

These strengths (automated data, real-time alerts, effective education, sustained adherence) addressed traditional follow-up limits and mechanistically supported better clinical outcomes (nutritional improvement, fewer complications, shorter RIOT).

### 3.6 Quality of life and adherence

Longitudinal assessment via EORTC QLQ-C30 showed the intervention group had significantly higher 12-month mean global health scores than the control group (72.4 vs 65.1; mean difference [MD] = 7.3, 95% CI: 1.1–13.5, P = 0.021, **Table 5**); functional domain results were consistent, with better overall health perception observed in the intervention group. The gastric cancer-specific QLQ-STO22 further showed the intervention group had greater reductions in eating difficulties, pain, and indigestion symptom scores (P<0.05), with lower gastrointestinal symptom burden and better quality-of-life scores observed in the intervention group.

**Table 5:** Comparison of quality of life, psychological status, and compliance outcomes at 12 months.

Outcome measure	Intervention (n=100)	Control (n=50)	Effect size (95% CI)	P-value
EORTC QLQ-C30				
Global health score (mean ± SD)	72.4 ± 10.8	65.1 ± 11.2	MD = 7.3 (1.1 to 13.5)	0.021
Functional dimensions (mean ± SD)	70.6 ± 12.0	63.7 ± 12.5	MD = 6.9 (0.5 to 13.3)	0.034
EORTC QLQ-STO22				
Symptom score (mean ± SD)	28.9 ± 8.4	34.7 ± 9.1	MD = -5.8 (-10.6 to -1.0)	0.018
Psychological Status				
Patients at risk of HADS (%)	8.00%	18.00%	OR = 0.39 (0.16 to 0.98)	0.04
Adherence				
≥80% prescription compliance (%)	73.00%	45.00%	OR = 3.28 (1.36 to 7.91)	0.008

MD, Mean Difference; OR, Odds Ratio; CI, Confidence Interval. EORTC QLQ-STO22 scores include dysphagia, pain, and dyspepsia; lower scores indicate fewer symptoms. HADS risk is defined as a subscale score ≥ 8.

For psychological status, HADS scores revealed the intervention group had a lower proportion of patients at anxiety/depression risk at 12 months (8.0% vs 18.0%, P = 0.04, **Table 5**). Lower HADS risk was observed in the intervention group, although patient-reported outcomes may have been influenced by greater contact and support and should therefore be interpreted cautiously.

In terms of adherence, the intervention group had a significantly higher proportion of patients achieving ≥80% nutritional prescription implementation (73.0% vs 45.0%; relative risk [RR] = 1.62, 95% CI: 1.15–2.28, P = 0.008, **Table 5**), aligning with the platform’s high task completion and education reach (Section 3.5). Higher prescription adherence was observed in the intervention group, possibly related to task reminders, automated monitoring, and personalized feedback—this adherence is key to nutritional benefits and long-term improvements in quality of life and psychological health.

**Figure 5:**
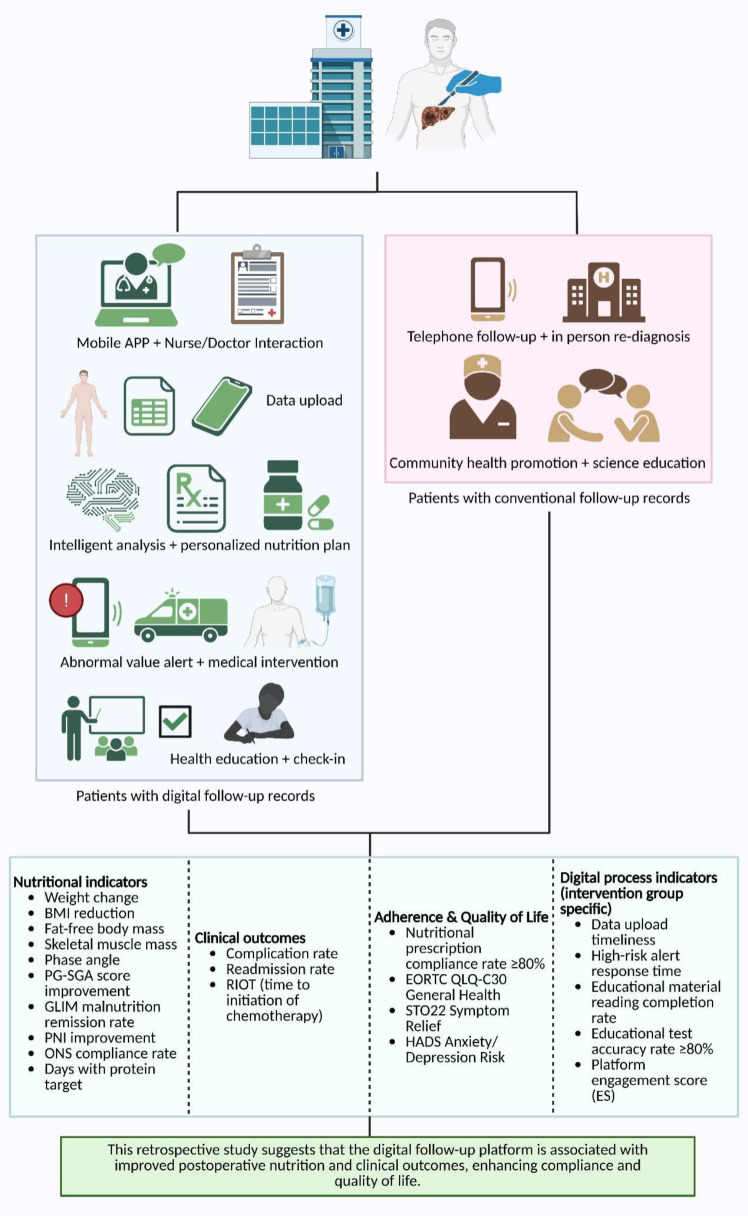
Overview of the digital Follow-up platform intervention model and outcome metrics.

**Figure 5** summarizes the digital follow-up platform-supported care framework and the corresponding outcome measures.

## 4 Discussion

Timely out-of-hospital malnutrition identification and correction remains a challenge (26, 27), thus this study aimed to address via a WeChat-based digital follow-up platform—overcoming traditional follow-up’s low frequency and fragmented data to boost nutritional management continuity and precision. In this study, the intervention group showed significant advantages in body composition vs. controls: at 12 months, mean weight and BMI gradually recovered (vs. continued decline in controls), with less skeletal muscle mass (SMM) loss (≈0.3 kg vs. 0.8 kg) and a ≈0.5° phase angle increase (indicating improved cellular membrane function) (25). These changes are clinically meaningful—excessive post-surgery weight (>15% at 1 month) or lean mass (>5% at 6 months) loss impairs therapy tolerance, with 91.7% treatment failure for the latter (28). Our findings align with Liu et al. (2023) [intensified personalized nutrition boosts lean mass/phase angle (29)] and Zeng et al. (2025) [perioperative support preserves weight/muscle and improves prognosis in digestive tract cancer (30)], reinforcing credibility.

Notably, the intervention group had a higher GLIM-defined malnutrition remission rate, with more patients transitioning to well-nourished. GLIM remission reflects overall nutritional improvement—GLIM-diagnosed malnutrition correlates with more complications and lower survival (31), so remission signals better prognosis. GLIM remission also aligned with PG-SGA improvements; a systematic review shows moderate agreement between GLIM and PG-SGA in cancer patients (sensitivity ≈71%, specificity 80%) (7), supporting GLIM as a PG-SGA alternative. Few digital studies use GLIM for efficacy evaluation, so our study fills this gap, offering a new dimension for digital health intervention assessment and suggesting future focus on GLIM to align with international nutrition advances.

Clinically, the intervention group had fewer postoperative complications, lower 30-day readmission rates, and shorter RIOT (time to adjuvant therapy) vs. controls—all linked to better nutrition. Malnutrition is an independent complication risk factor [e.g., hypoalbuminemia/sarcopenia raise wound infection/respiratory failure risk (32)]; early nutritional risk identification and intervention in the platform-supported care pathway may have helped maintain nutritional reserves and may be associated with fewer severe complications. This aligns with literature: GLIM-malnourished patients have 57% higher complication risk (7), and GLIM diagnosis predicts higher 30-day readmission risk (33)—enhanced support mitigates this. Shorter RIOT means earlier adjuvant therapy eligibility, critical for gastric cancer survival; malnutrition often delays/discontinues therapy (34), but the intervention’s reduced weight/muscle loss let patients meet chemotherapy physical requirements sooner—consistent with Jung et al.’s finding that perioperative nutrition boosts chemotherapy tolerance (35). Overall, the platform-supported care pathway was associated with better recovery and care continuity, although the independent contribution of the digital component itself cannot be determined from this study design.

Process metrics in the intervention group showed the platform’s strengths: 89.0% timely data submission (exceeding conventional self-report), higher protein intake compliance and task completion vs. controls—capabilities traditional follow-up lacks. Conventional oral nutritional supplement (ONS) compliance in gastric cancer is as low as 24.7% (36), but our digital follow-up platform-supported nutritional care achieved 73% nutritional prescription adherence (vs. 45% in controls) via structured engagement and reminders—aligning with Roberts et al.’s finding that technology enhances participation (37). The platform’s 14-hour median alert response time (vs. traditional multi-day delays) boosts care efficiency, corroborating evidence that structured digital nutrition interventions improve dietary compliance and quality of life (38), and shifting patients from passive to active care participants—which may be relevant to long-term outcomes, although patient-reported measures should be interpreted cautiously because participants were not blinded.

Our analysis suggests that greater platform engagement was associated with more favorable outcomes within the platform-supported care pathway, although this should not be interpreted as isolating the independent effect of the digital tool itself. High-engagement patients had greater phase angle improvement and less skeletal muscle wasting, showing a clear dose-response between platform use and clinical benefit—consistent with Furness et al.’s systematic review that eHealth efficacy in cancer rehabilitation correlates with engagement (39).

Mediation analysis revealed protein intake attainment mediated 36% of the platform’s effect on nutritional recovery; active platform use improved high-protein prescription compliance, addressing core nutritional deficits—aligning with Kim et al.’s finding that protein optimization is critical for muscle preservation in older adult surgical patients (40). After multivariate adjustment, engagement independently predicted nutritional outcomes; SHAP modeling further confirmed platform-recorded behavioral metrics (protein intake, weight documentation, symptom reporting) as key predictors of recovery, translating digital engagement into clinically actionable insights beyond traditional statistics.

The WeChat-integrated platform showed strong usability and acceptability across demographics. China’s widespread WeChat ecosystem [especially used by middle-aged and older adults for health management (41)] supported deployment; its intuitive interface enabled adoption by most patients/caregivers—even those with low tech proficiency or in resource-limited settings—with supplemental telephone guidance bridging digital literacy gaps. While the platform’s minimal requirements (smartphone and internet) generally support accessibility, implementation in regions with lower digital access or among populations with limited digital literacy may face significant logistical barriers. Future scaling in community-based settings may require simplified, low-bandwidth interfaces or a hybrid model combining digital alerts with existing offline community health services to bridge the digital divide.

The intervention’s replicability stems from evidence-based components: GLIM-aligned assessment, personalized protein prescriptions, and graded alerts all match international guidelines (42). Other institutions can adapt it via nutritional support teams and context-specific tweaks. With healthcare digitalization, the platform could expand to perioperative care and chronic diseases, aiding resource redistribution; future integration with national insurance/tiered healthcare may boost adoption, while voice-command and simplified workflows will better serve older adult users.

Despite the promising findings, this study has several limitations that warrant consideration. First and foremost, its retrospective and non-randomized design is an inherent source of potential bias. Although we employed robust statistical methods, specifically Inverse Probability of Treatment Weighting (IPTW), to balance measured baseline covariates between the intervention and control groups, the possibility of residual confounding due to unmeasured or unrecorded factors cannot be entirely ruled out. For instance, due to the retrospective nature of this study, we were unable to systematically collect several factors such as baseline motivation, and nutritional counseling intensity, thus residual confounding from these unmeasured variables cannot be ruled out. Future prospective studies should incorporate these factors to better isolate the independent effect of digital health interventions and to identify subgroups that may benefit most from such approaches. Secondly, the single-center design within a high-volume tertiary academic hospital limits the external validity of our findings. The generalizability to community hospitals may be restricted by the absence of specialized nutritional support teams and the intensive nursing resources available in our center, which were critical for the platform’s high-frequency monitoring and rapid alert response. The high adherence rates observed might reflect the specialized academic environment; consequently, the model’s success in community settings or regions with restricted digital infrastructure remains to be validated, highlighting the need for context-specific adaptations of the digital follow-up workflow. Thirdly, the outcomes assessors were not blinded to the group allocation, which could have introduced measurement bias, particularly for patient-reported outcomes and the clinical adjudication of certain complications. Additionally, because the sample size was determined by the available retrospective cohort rather than a comprehensive *a priori* calculation for all endpoints, the statistical power to detect significant differences in less frequent secondary outcomes (such as specific complication subtypes) may be limited, which inherently weakens the strength of inference for these secondary measures. Furthermore, the 12-month follow-up period, while sufficient for assessing nutritional and short-term clinical outcomes, is too short to evaluate the long-term impact of the digital follow-up platform-supported nutritional care on overall survival and cancer recurrence. Finally, a key limitation of our study design is the inability to isolate the independent contribution of the digital platform from the structured nutritional care itself. Because the intervention group received proactive, intensive nutritional support (including ONS/enteral nutrition and corrective interventions) delivered via the platform, while the control group received only minimal post-discharge management, the comparison is essentially between a “structured, proactive nutritional care model” and “standard minimal care.” Therefore, we cannot definitively determine whether the observed benefits are primarily attributable to the technological delivery mechanism (the digital platform) or the enhanced nutritional protocol itself. Furthermore, the digital platform introduced a “novelty effect,” and the observed improvements might be influenced by the increased attention received by the intervention group; the long-term sustainability of patient engagement remains to be evaluated.

Future research should address these limitations through prospective, multi-center, randomized controlled trials with longer follow-up periods and efforts to blind outcome assessors. Incorporating objective measures of socioeconomic status and patient activation would also help to clarify the true independent effect of the digital follow-up platform-supported nutritional care.

## 5 Conclusion

This retrospective controlled study suggests that a structured, proactive nutritional care model, supported by a digital follow-up platform, was associated with better nutritional outcomes in patients following radical gastrectomy for gastric cancer, as compared to conventional follow-up. The intervention group demonstrated more favorable body composition parameters, a higher rate of GLIM-defined malnutrition remission, and lower incidences of complications and readmissions. In addition, a shorter time to initiation of adjuvant therapy was observed. The platform allowed for detailed tracking of care processes and revealed a relationship between patient engagement and clinical outcomes, suggesting a potential role for adherence in the effectiveness of digital follow-up platform-supported nutritional care. By incorporating standardized GLIM assessment, process monitoring, and clinical endpoints, this model addresses a recognized gap in post-discharge nutritional management. Its relatively low implementation requirements and potential for scalability may support broader application in various healthcare settings. Overall, this digital follow-up approach may represent a structured and promising framework for postoperative nutritional care in oncology.

## Data Availability

The raw data supporting the conclusions of this article will be made available by the authors, without undue reservation.

## Supplementary material

Supplementary Table S1 PSM-matched baseline characteristics and main outcomes.

Supplementary Figure S1 Forest plot of subgroup analyses.
