# Supplementary material for: Structured proactive nutritional care delivered via a digital follow-up platform is associated with better postoperative outcomes in gastric cancer patients after radical gastrectomy
Source: Front Oncol. 2026 Apr 21;16:1734663. doi: 10.3389/fonc.2026.1734663 (PMC13138984; doi:10.3389/fonc.2026.1734663)

### Supplementary Material S1: Nutritional Prescription Decision Algorithm for the Digital Platform

**Overview:** This algorithm is based on the **ESPEN Guidelines on Nutrition in Cancer Patients**. The initial draft is automatically calculated by the platform backend and is subsequently reviewed and finalized by clinical nutrition nurses.

#### Stage 1: Automatic Data Input

#### Baseline Data: Sex, age, current body weight (W_curr_), height, and BMI.

**Metabolic Assessment:** Based on the Harris-Benedict equation or the platform’s built-in Basal Metabolic Rate (BMR) model.

**Nutritional Risk:** Results of the Global Leadership Initiative on Malnutrition (GLIM) assessment (combining phenotypic and etiologic criteria).

#### Stage 2: Target Calculation Algorithm

The platform automatically generates target values according to the following logic:

**Total Energy Expenditure (TEE):**

**Formula:** 25-30 kcal/kg/day (adjusted based on physical activity levels and stress factors).

**Total Protein Intake:**

**Formula:** 1.2 - 1.5 g/kg/day.

**Non-protein Calorie Ratio:** The ratio of carbohydrates to fats is typically set at 50:50 or 60:40.

#### Stage 3: Prescription Decision Tree

Classification and intervention levels are determined by the difference between the patient's 24-hour dietary intake (I_{diet}) and the calculated target value (T):

**Path A: Adequate Dietary Intake (I_diet_≥ 80% T)**

**Recommendation:** Maintain the current dietary plan.

**Content:** Strengthen education on high-protein diets and provide stage-specific meal plans.

**Path B: Inadequate Dietary Intake (50%≤I_diet_< 80% T)**

**Recommendation:** Initiate Oral Nutritional Supplements (ONS).

**Content:** Add ONS 2 - 3 times daily (approximately 400 - 600 kcal) and adjust the supplement type based on the patient's taste preferences.

**Path C: Severely Inadequate Intake or Severe Gastrointestinal Symptoms (I_diet_< 50% T)**

**Recommendation:** Intensified intervention or consideration of Enteral Nutrition (EN).

**Content:** The nurse conducts a video consultation within 24 hours to adjust the patient to a full liquid/semi-liquid diet. If symptoms do not improve within 3 days, the patient is advised to return to the hospital for assessment of enteral access (e.g., jejunostomy tube).

#### Stage 4: Nurse Review & Feedback Loop

**Review Points:** Nurses evaluate potential risks or contraindications, such as refeeding syndrome, by reviewing the patient's latest laboratory indicators (e.g., serum phosphorus, electrolytes, and renal function).

**Publishing:** The final verified prescription is pushed to the patient-side App/mini-program in a clear, graphical format.

**Monitoring:** The platform monitors daily patient check-ins regarding prescription implementation and automatically calculates the compliance rate.

**Supplementary Material S2: Assessment of Covariate Balance**

To evaluate the effectiveness of the Inverse Probability of Treatment Weighting (IPTW), absolute standardized mean differences (ASMD) were calculated for all baseline covariates before and after weighting. Figure S2 (Love Plot) illustrates that while several variables (e.g., BMI, preoperative PG-SGA) showed imbalances in the unweighted cohort (ASMD > 0.1), the application of stabilized weights successfully reduced all ASMDs to below the 0.10 threshold. Furthermore, the variance ratios for all continuous covariates post-weighting were close to 1.0, indicating that the intervention and control groups achieved robust distributional balance, thereby minimizing potential selection bias in subsequent outcome analyses.


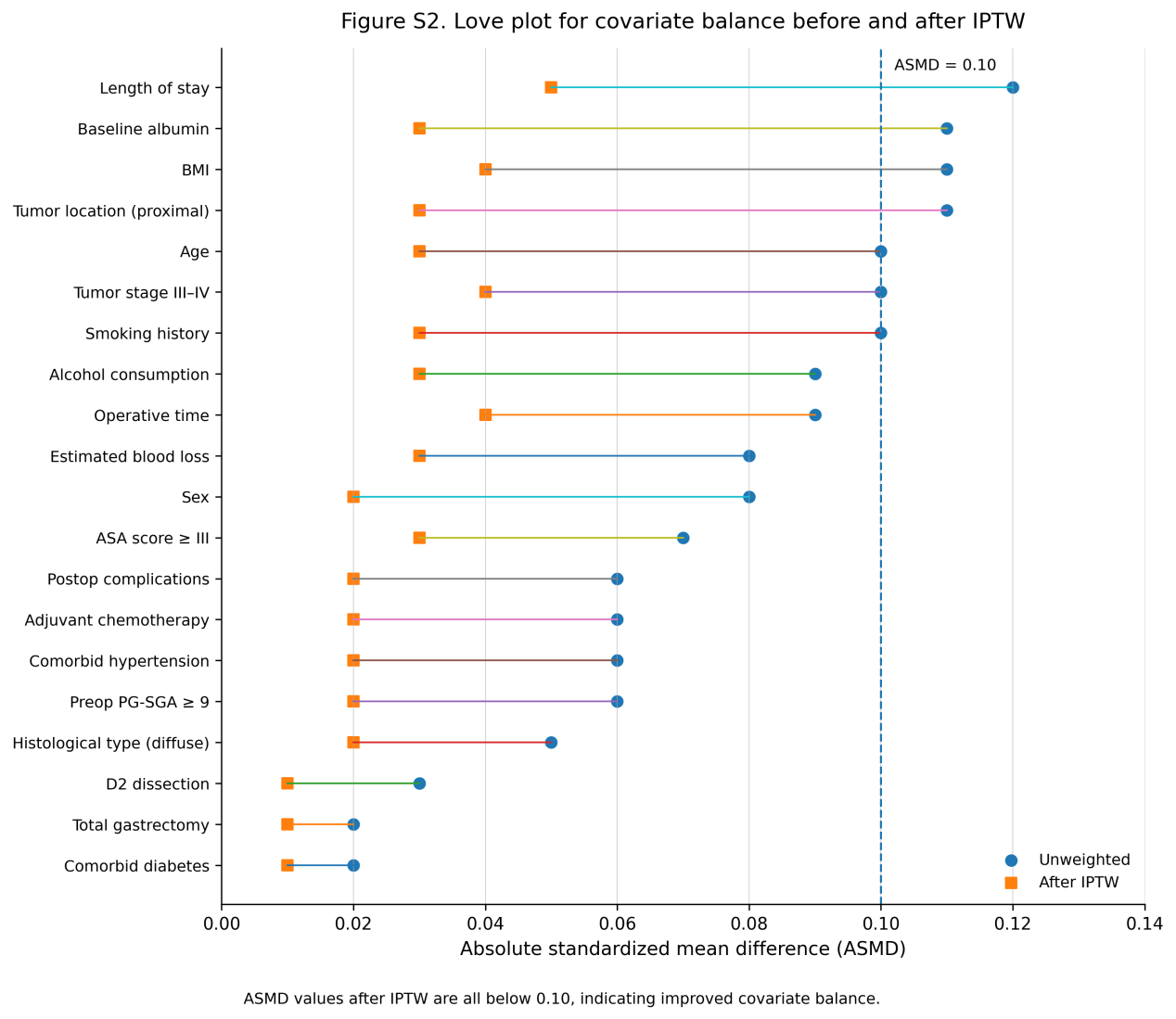

Supplement: Supplementary Table S1 — PSM-matched baseline characteristics and main outcomes. [file DataSheet1.docx]
