# Supplementary material for: Structured proactive nutritional care delivered via a digital follow-up platform is associated with better postoperative outcomes in gastric cancer patients after radical gastrectomy
Source: Front Oncol. 2026 Apr 21;16:1734663. doi: 10.3389/fonc.2026.1734663 (PMC13138984; doi:10.3389/fonc.2026.1734663)
Supplement: Supplementary Figure S1 — Forest plot of subgroup analyses. [file Supplementaryfile1.docx]

**Table S1. PSM-matched baseline characteristics and main outcomes**

| **Characteristic / outcome** | **Intervention group (n=50)** | **Control group (n=50)** | **P value** | **SMD** |
| --- | --- | --- | --- | --- |
| **Baseline characteristics** | | | | |
| Age, years (mean ± SD) | 59.3 ± 8.5 | 59.8 ± 8.9 | 0.78 | 0.06 |
| Sex, male, n (%) | 31 (62.0) | 30 (60.0) | 0.84 | 0.04 |
| Body mass index, kg/m² (mean ± SD) | 22.3 ± 2.7 | 22.2 ± 2.6 | 0.87 | 0.03 |
| Smoking history, n (%) | 19 (38.0) | 18 (36.0) | 0.84 | 0.04 |
| Alcohol consumption, n (%) | 18 (36.0) | 17 (34.0) | 0.83 | 0.04 |
| Comorbid diabetes, n (%) | 9 (18.0) | 9 (18.0) | >0.99 | 0 |
| Comorbid hypertension, n (%) | 14 (28.0) | 14 (28.0) | >0.99 | 0 |
| ASA score ≥ III, n (%) | 11 (22.0) | 12 (24.0) | 0.81 | 0.05 |
| Preoperative PG-SGA score ≥ 9, n (%) | 7 (14.0) | 8 (16.0) | 0.78 | 0.05 |
| Tumor stage (AJCC 8th), III-IV, n (%) | 24 (48.0) | 25 (50.0) | 0.84 | 0.04 |
| Type of gastrectomy, total, n (%) | 19 (38.0) | 19 (38.0) | >0.99 | 0 |
| Adjuvant chemotherapy, n (%) | 28 (56.0) | 27 (54.0) | 0.84 | 0.04 |
| Baseline albumin, g/L (mean ± SD) | 39.6 ± 4.4 | 39.3 ± 4.5 | 0.73 | 0.07 |
| **Main outcomes at 12 months** | | | | |
| GLIM nutritional remission, n (%) | 29 (58.0) | 17 (34.0) | 0.016 | 0.49 |
| Overall nutrition-related complications, n (%) | 11 (22.0) | 20 (40.0) | 0.049 | 0.39 |
| Readmission due to malnutrition or related complications, n (%) | 5 (10.0) | 12 (24.0) | 0.059 | 0.39 |
| Time-to-RIOT, days (median [IQR]) | 32 [24-42] | 45 [35-58] | 0.028 | — |
| RIOT rate, n (%) | 43 (86.0) | 37 (74.0) | 0.129 | 0.3 |

**
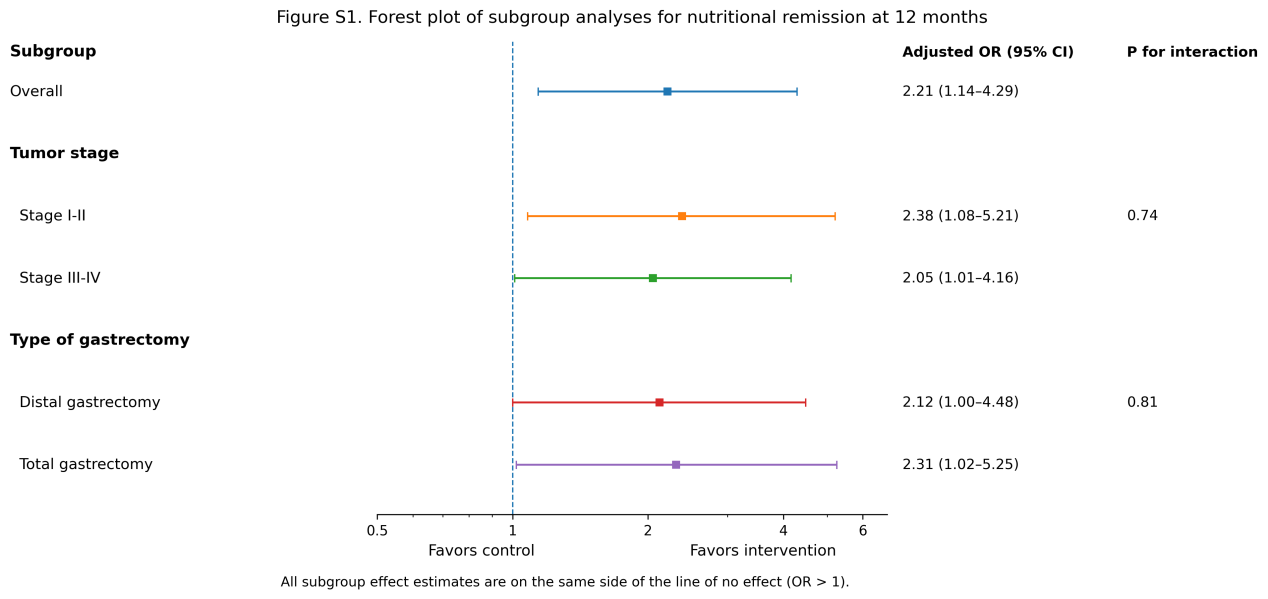
**

**Figure S1. Forest plot of subgroup analyses**
